# Aquaculture Strategy and Genetic Diversity of *Argopecten irradians concentricus* in Beibu Gulf, China

**DOI:** 10.3390/biology14081103

**Published:** 2025-08-21

**Authors:** Qishuai Wang, Jie Feng, Yanping Qin, Ying Pan

**Affiliations:** 1 Key Laboratory of Aquatic Healthy Breeding and Nutrition Regulation of Guangxi Universities, College of Animal Science and Technology, Guangxi University, Nanning 530004, China; 2 CAS Key Laboratory of Tropical Marine Bio-Resources and Ecology, South China Sea Institute of Oceanology, Chinese Academy of Sciences, Guangzhou 510301, China; 3 Guangdong Provincial Key Laboratory of Applied Marine Biology, Innovation Academy of South China Sea Ecology and Environmental Engineering, Guangzhou 510301, China

**Keywords:** *Argopecten irradians concentricus*, breeding population, stocking density, genetic diversity, Beibu Gulf

## Abstract

*Argopecten irradians concentricus* has become one of the pillar industries in the aquaculture of the Beibu Gulf since it was introduced into China in 1991. This study examined how stocking density and culture site affects growth in breeding populations, compared their growth performance and genetic diversity within control populations, and identified optimal culture locations for *A. i. concentricus* in the Beibu Gulf. The environmental investigation results revealed that among the three aquaculture sites of Beihai (BH), Qinzhou (QZ) and Fangchenggang (FCG), the fluctuation ranges of salinity, pH, and dissolved oxygen at the BH site were relatively narrower. The sum of all algal genus abundances of the three sites were 155,370 cells∙L^−1^, 931 cells∙L^−1^, and 47,957 cells∙L^−1^, respectively. *Chaetoceros* was the sole dominant algal genus in both BH and FCG, while *Pleurosigma* was the only dominant genus in QZ. The experimental results of growth demonstrated a significant negative correlation between growth rate and stocking density (*p* < 0.05). The mortalities of the QZ populations were significantly higher than those of the BH and FCG populations (*p* < 0.05). In comparison with the control populations, the breeding populations exhibited better growth performance but lower genetic diversity. FCG is a suitable location for cultivating the breeding population of *A. i. concentricus*. The findings of this study can serve as a reference for further understanding of the aquaculture strategy and genetic diversity of *A. i. concentricus* in the Beibu Gulf, China.

## Simple Summary

Since its introduction to China in 1991, *Argopecten irradians concentricus* has become a pillar species in Beibu Gulf aquaculture. This study examined the effects of stocking density and culture site on growth in breeding populations, compared their growth performance and genetic diversity within control populations, and identified optimal culture locations. Environmental analysis revealed Beihai (BH) exhibited narrower fluctuations in salinity, pH, and dissolved oxygen; total algal abundances were 155,370 cells∙L^−1^ (BH), 931 cells∙L^−1^ (QZ), and 47,957 cells∙L^−1^ (FCG), with *Chaetoceros* dominant in BH and FCG, and *Pleurosigma* dominant in QZ. Growth experiments demonstrated a significant negative correlation between growth rate and stocking density, while QZ populations exhibited significantly higher mortality. Breeding populations showed superior growth performance, but lower genetic diversity compared to controls. FCG was identified as a suitable site for cultivating breeding populations, with these findings providing a reference for understanding the species’ aquaculture management strategies and genetic diversity in the region.

## 1. Introduction

*Argopecten irradians concentricus*, which is naturally distributed along the Atlantic coasts of the United States and the Gulf of Mexico, is a eurythermal species with a temperature range of 13–33 °C [1]. It was introduced to China in 1991 and since been widely cultured in southern China [1]. The Beibu Gulf, located in the northwest of the South China Sea, is a major aquaculture area for mollusk aquaculture [2,3]. In 2002, *A. i. concentricus* began to be promoted and bred in the Beibu Gulf and achieved remarkable success [4]. However, the profitability of the scallop industry decreased sharply due to its high mortality, slow growth, and frequent occurrence of diseases, presumably resulting from severe inbreeding in the last decade [5]. To address this and with the purpose of genetic improvement, Liu et al. [6] initiated selective breeding on it in 2007. After continuous generations of selection and breeding, a genetically stable breeding population was obtained.

Currently, studies on *A. i. concentricus* genetic breeding [7,8], artificial breeding [5,9], the effects of environmental factors on individuals [10,11], and the development and characterization of microsatellite loci [12,13] have been reported. These studies have provided foundations for the cultivation of *A. i. concentricus* in the Beibu Gulf of China. For the aquaculture of *A. i. concentricus* in China, the feeding environment and stocking mode are important factors influencing productive performance. Bivalves, which feed on phytoplankton, zooplankton, and suspended solids, are significantly influenced by these food sources in terms of their survival and growth [14]. Previous studies on the stocking density of *Meretrix lyrata* [15], *Haliotis discus hannai* Ino [16], *Mizuhopecten yessoensis* [17], *Chlamys farreri* [18], and *Pinctada martensi* [19] have revealed that stocking density has significant impacts on the growth rate and mortality of shellfish, and a reasonable density can greatly enhance breeding efficiency. Nevertheless, there has been no report on the optimum stocking density of *A. i. concentricus* in the Beibu Gulf of China. Exploring appropriate breeding environments and stocking densities can further promote the development of *A. i. concentricus* aquaculture.

This study aims to assess the effects of stocking density and sites on the growth traits of the breeding population of *A. i. concentricus*. Additionally, we compared the growth traits and genetic diversity between the breeding population and the control populations to assess the growth performance of the breeding population. The results of this study may offer a theoretical basis for the directional and rational expansion of the production scale of the *A. i. concentricus* industry in the Beibu Gulf.

## 2. Materials and Methods

### 2.1. Experimental Sites and Scallops

The experimental sites set along the coast of the Beibu Gulf in China, were Beihai (BH, 21°01′ N, 109°06′ E), Qinzhou (QZ, 21°40′ N, 108°42′ E), and Fangchenggang (FCG, 21°31′ N, 108°14′ E), respectively. The scallop breeding population employed in this experiment comprised the eighth selected generation (G8) derived through successive selective breeding. In contrast, the three control populations were founded via artificial breeding using non-selected scallop broodstock cultured at the three respective locations. The breeding group and the control group were jointly bred in a nursery in Zhanjiang, Guangdong. After a specific time interval, they were gathered and transferred to the experimental sites concurrently.

### 2.2. Growth Experiment

The effect of different culture conditions on growth in different scallops’ strains at the nursery stage and the adult stage, were compared. The stocking density gradient referenced the research and production practices of other cultured scallop species in China [20,21]. The nursery culture stage took place from December 2015 to February 2016. At each of the given sites, both the breeding group and the control group were cultivated in cylindrical lanterns furnished with six layers of substrates [14]. The stocking densities were set at 100, 200, and 300 scallops per layer, with six replicates established for each density. The hanging culture depth was around 1.5 m beneath the water surface, and the hanging culture lanterns were spaced approximately 1 m apart. Upon the conclusion of the nursery culture stage, scallops possessing a shell length and shell height of approximately 30 mm were chosen for the adult stage aquaculture experiment. The stocking densities for this stage were 30, 45, and 60 scallops per layer, while the other conditions remained consistent with those of the nursery culture stage. The adult culture stage was carried out from March 2016 to June 2016. On a monthly basis during the experimental period, all scallops were counted, weighed, and their shell height, shell length, and shell width were measured. Subsequently, they were continuously reared until the end of the experiment. Additionally, one extra lantern with the same density as the experimental group was utilized to replace the dead scallops [14,22].

### 2.3. Environmental Factor and Phytoplankton Analysis

During the aquaculture period, monthly investigations were carried out on the environmental factors and phytoplankton at the three sites. The specific measurement methods for pH, dissolved oxygen, and other parameters, as well as the identification and counting methods of phytoplankton, were in accordance with those described by Wei et al. [14].

### 2.4. DNA Isolation and PCR Amplification

**Figure 1:**
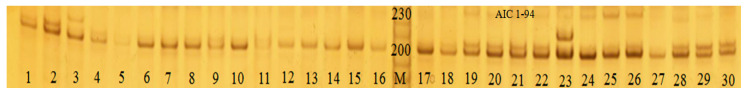
Visualization of amplification products from microsatellite locus AIC 1-94 via polyacrylamide gel electrophoresis (representative subset).

Upon the completion of the growth experiment, 30 scallop muscle samples were randomly collected from each of the six populations, respectively, and then preserved in absolute ethanol. Genomic DNA was extracted using the traditional proteinase K and phenol-chloroform extraction method [23]. The primers were cited from Hu et al. [13], and the basic information is presented in Table 1. The total volume of PCRs was 10 μL, which consisted of 5 μL Taq Mix (Trans Gen, Beijing, China), 2 μL ddH_2_O, 1 μL of each primer (10 μM), and 1 μL DNA. The reaction procedure was as follows: initial denaturation at 94 °C for 3 min, followed by 35 cycles of denaturation at 94 °C for 45 s, annealing at a primer-specific temperature for 45 s, extension at 72 °C for 45 s, and a final extension at 72 °C for 5 min. Non-denaturing polyacrylamide gel (8%) was employed for the electrophoresis detection of the PCR amplification product, with comparison to the 10 bp DNA ladder (Figure 1). After silver nitrate staining, the results were screened using a gel scanner (Bio-Rad, Hercules, CA, USA).

### 2.5. Statistical Analysis

All data were presented in the form of mean ± standard deviation. The statistical analyses were carried out using SPSS Statistics 26.0. Prior to performing the analyses, normality and variance homogeneity of all variables were tested by using Kolmogorov–Smirnov and Levene’s tests, respectively. One-way ANOVA was then conducted, followed by Turkey multiple comparison tests, to compare the growth parameters of the two strains under different stocking densities, as well as to compare the environmental factors at different experimental sites. A *p*-value of less than 0.05 indicated a significant difference. The correlation analysis of growth traits and stocking density were conducted by Pearson correlation analysis and a two-sided *t*-test.

Dominant species in the microalgae community are defined as those with a dominance greater than 0.2. The calculation formula of dominance was expressed as: $Y=\frac{n_{i}\times f_{i}}{N}$, where *Y* represents dominance, *N* represents the total cell abundance of the community, *n_i_* represents the cell abundance of *i*-th species in the community, and *f_i_* represents the frequency of the *i*-th species occurring at each site in the community [14].

Electrophoretic analysis was carried out with Quantity One, and following manual calibration, the size of bands was estimated. The number of alleles (*N_A_*), observed heterozygosity (*H_O_*), and expected heterozygosity (*H_E_*) were computed using Popgen 32.0. The polymorphism information content was determined by Cervus 3.0. MEGA7.0 and was utilized to construct the clustering tree based on the genetic distance between each population.

## 3. Results

### 3.1. Effect of Stocking Density, Site, and Strain on the Growth of A. i. concentricus

The harvest data of the nursery and adult culture stages were summarized in Table 2. In general, irrespective of the specific aquaculture site and strain, a significant negative correlation was observed between growth rate and stocking density (*p* < 0.05). During the nursery culture stage, only when the stocking density was 100 scallops per layer did all the growth traits turn out to be significantly lower than those of the control population (*p* < 0.05). In the case of the breeding population cultured in FCG, only when the stocking density was 200 scallops per layer were all the growth traits significantly higher than those of the control population (*p* < 0.05). For the breeding population cultured in QZ, only a certain trait under a specific stocking density was different from that of the control population. For instance, when the stocking density was 200, the breeding population was significantly lower than the control population in terms of body weight (*p* < 0.05). Regarding the harvest data of the adult stage, under the same site conditions and stocking density, all growth traits of the breeding groups were significantly higher than those of the control groups. (*p* < 0.05). The breeding population exhibited the fastest growth in FCG and the slowest growth in QZ.

### 3.2. Effect of Different Months on the Mortality of A. i. concentricus in Adult Culture Stage

**Figure 2:**
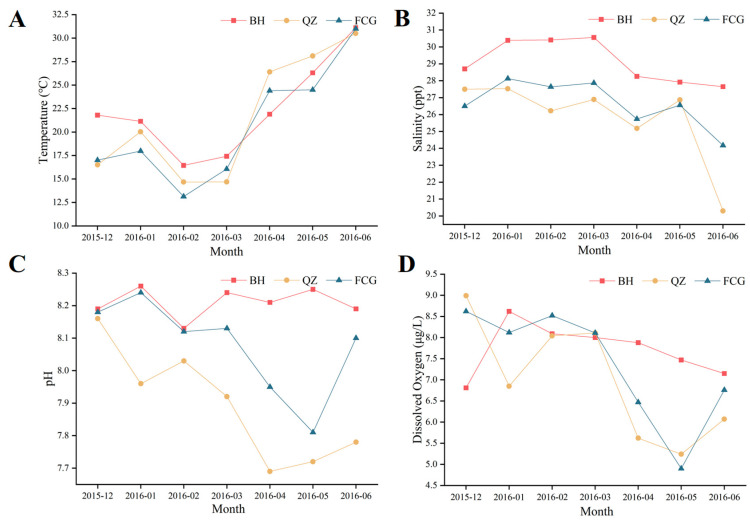
The variations in (**A**) temperature, (**B**) salinity, (**C**) pH and, (**D**) dissolved oxygen at the three cultured sites from December 2015 to June 2016.

Table 3 presents the mortality situation of scallops in different months during the adult culture stage. For sites, strains, and stocking densities, a sharp increase in mortality was observed in June. This might be attributed to the relatively high seawater temperature in June, as depicted in Figure 2A. Among the different populations, the mortalities of the QZ populations were significantly higher than those of the BH and FCG populations (*p* < 0.05). For the same site and the same stocking density, no significant differences in mortality were detected between the breeding groups and the control groups.

### 3.3. Seawater Quality and Plankton at the Three Sites

Figure 2 illustrates the variations in seawater temperature, salinity, pH, and dissolved oxygen, at the three cultured sites. No significant differences in temperature and dissolved oxygen were found among the three sites, whereas significant differences were present in salinity and pH (*p* < 0.05). In comparison with QZ and FCG, the fluctuation ranges of salinity, pH, and dissolved oxygen at the BH site were relatively narrower.

A total of twenty-eight, twenty-nine, and twenty-seven genera of phytoplankton were identified at the BH, QZ, and FCG sites, respectively (Table 4). The total abundances of all algal genera at the three aquaculture sites were 155,370 cells∙L^−1^, 931 cells∙L^−1^, and 47,957 cells∙L^−1^, respectively. *Chaetoceros* was the most abundant algal genus in both BH and FCG and was the only dominant genus. Meanwhile, *Pleurosigma* was the only dominant genus in QZ. The algae with a 100% occurrence frequency at the aquaculture sites were as follows: *Chaetoceros* in BH, *Coscinodiscus*, *Pleurosigma*, *Navicula*, *Nitzschia* in QZ, and *Coscinodiscus*, *Thalassionema*, *Pleurosigma*, *Pinnularia*, *Ceratium furca* in FCG.

### 3.4. Genetic Variability of Six Scallop Cultured Populations

**Figure 3:**
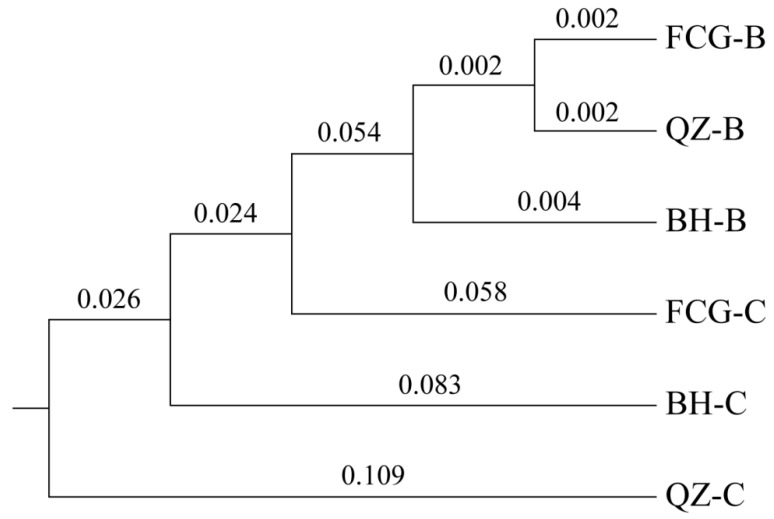
The UPGMA clustering tree of six scallop cultured populations based on Nei’s genetic distance. B, Breeding population of *A. i. concentricus*; C, Control population of *A. i. concentricus*.

Table 5 presents that the average number of alleles in six scallop populations spanned from 2.80 (BH-B or QZ-B) to 4.40 (BH-C). The average number of effective alleles, average observed heterozygosity, and expected heterozygosity varied from 1.82 (QZ-B) to 3.04 (BH-C), 0.37 (BH-B) to 0.46 (QZ-C), and 0.44 (QZ-B) to 0.62 (BH-C), respectively. With the exception of the control populations at two sites, BH-C and FCG-C, which exhibited high genetic diversity (PIC > 0.5), all other populations displayed moderate genetic diversity (0.25 < PIC < 0.5) [24]. The UPGMA clustering results indicated that the breeding populations BH-B, QZ-B, and FCG-B from the three cultured sites were initially clustered into one group due to their closer genetic distance. Subsequently, the three control populations, FCG-C, BH-C, and QZ-C, were clustered into the group in sequence (Figure 3).

## 4. Discussion

### 4.1. Effects of Different Stocking Densities on Growth Performance

The scallop production performance is influenced by numerous factors. These include water temperature [25], salinity [26], attachment organisms [27], aquaculture capacity [28], and culture methods [29], among others. Multiple reports have demonstrated that stocking density significantly impacts the growth of aquatic organisms. High stocking density can lead to food competition, slower growth, and significant size differences [20,21,30]. Due to high stocking density, aquaculture animals have adverse effects on the environment, such as lower dissolved oxygen (DO) and excessive ammonium-nitrogen (NH_4_–N) [31]. Simultaneously, the deteriorated environment, in turn, affects the growth and survival of aquatic organisms, resulting in a reduced growth rate and increased mortality [31]. Some studies have discovered that mortality may not be affected within a certain density range; however, if the stocking density exceeds a threshold, mortality will rise [32]. Marshall and Dunham [33] conducted a study on the survival rates of *Crassostrea gigas* and *Venerupis philippinarum* under different stocking densities and found that at low stocking densities, the mortality rate was low, and there were no significant differences among different stocking densities. In this study, growth experiments on *A. i. concentricus* were carried out at different densities in various locations. It was found that growth performance decreased remarkably with the increase in stocking density. This is in line with the research findings in *Salmo salar* [34], *Litopenaeus vannamei* [30], *Pationopecten yessoensis* [20], *Haliotis discus hannai* Ino [16], and *Crassostrea nippona* [26].

### 4.2. Effects of Different Sites on Growth Performance

Different environments have a substantial impact on the growth of scallops. The *A. i. concentricus* is classified as a eurythermal but stenohaline bivalve, with an optimal temperature range of 27.5–30 °C and an optimal salinity range of 28.7–31.3 ppt for its growth [35]. Qinzhou and Fangchenggang are close to the shore and near river estuaries, making them susceptible to the impacts of rainfall and inland runoff. They are particularly prone to abrupt declines in water salinity caused by heavy rainfall. In contrast, the experimental site in Beihai is far from the mainland and thus, less affected. In this study, the mortality rate of the Qinzhou population, regardless of high or low density, was significantly higher than that of the populations in Beihai and Fangchenggang in June, which may be associated with the abrupt decline in salinity at the Qinzhou aquaculture site during this month. The content of organic carbon in water has a significant influence on the growth of filter-feeding shellfish [36], and algae are also a major food source for filter-feeding shellfish [37]. The study found that the phytoplankton abundance in the Qinzhou aquaculture site was significantly lower than that in the Beihai and Fangchenggang aquaculture sites, which may be one of the reasons for the poor growth performance of the population in this region. It is noteworthy that among the three aquaculture sites, the Beihai site exhibits the most stable and optimal water quality for the growth of *A. i. concentricus* as well as the highest phytoplankton richness; however, its growth performance is slightly inferior to that of the Fangchenggang site, and the underlying reasons for this phenomenon require further investigation.

### 4.3. Comparison of the Genetic Diversity of Populations

Heterozygosity is a crucial indicator for assessing the genetic diversity of a population [38]. The higher the heterozygosity, the greater the degree of genetic variation. Expected heterozygosity represents the theoretical frequency of heterozygotes when a population is in an equilibrium state. Compared with observed heterozygosity, it can more accurately reflect the genetic diversity level of a population [39]. In this study, in comparison with the three control populations of *A. i. concentricus*, the expected heterozygosity (*H_E_*) and polymorphic information content (*PIC*) of the selected breeding populations were slightly reduced, suggesting a decrease in the genetic diversity of the selected population during the continuous artificial breeding process.

## 5. Conclusions

In summary, a significant negative correlation exists between growth rate and stocking density. The breeding populations display better growth performance despite lower genetic diversity in comparison to the control populations. Among the three aquaculture sites, the seawater environment in Beihai exhibits relatively less fluctuation and possesses the highest microalgae diversity. In contrast, the algal genus abundance in Qinzhou is the lowest, and its mortality rate is the highest. The mortality of the Beihai populations and the Fangchenggang populations during the adult culture period are similar. Based on the growth rate, Fangchenggang is determined to be the optimal site for cultivating the breeding populations of *A. i. concentricus*. The optimal stocking density of scallops is contingent upon the overall cultivation strategy and is consequently influenced by multiple factors such as the desired final market product, market size, and types of growth techniques. Further research remains necessary.

## Figures and Tables

**Table 1:** Information on ten pairs of microsatellite primers.

Locus	Repeat Motif	Primer Sequences	Tm (°C)	Size (bp)
AIC1-50	(GT)_17_	F: AGGGTGGTGAAGCAGGGAC R: GGATTACGCCAGCTATTTAGGTG	55	320–353
AIC1-94	(GT)_16_	F: TTTATGAGAACAGGCACAGC R: TGGACATGACATGATTACGC	51	151–182
AIC2-9	(GT)_16_	F: CCTTGTTATCTGCCATTTCG R: GACATGATTACGCCCAGCTAT	51	646–677
AIC2-77	(TG)_18_	F: TACAATGAACAGGGAAAGTCGG R: CGTACGCCAGCTATTTAGGTGA	55	190–225
AIC3-45	(CA)_5_ … (TG)_5_ … (TG)_16_	F: ACGACAGGTTTCCCGACTT R: TTAACGCCAGGGTTTTCC	53	277–391
AIC3-47	(GT)_16_	F: CTCATCCAGAGGACCAACTT R: GGTGATTACGCCAGCTATTT	51	430–461
AIC4-74	(CA)_35_	F: TGACCATGATTACGCCAAGT R: GGCACTCGTCGTAGTAGTAACAT	54	113–182
AIC4-78	(CA)_17_	F: ATTACGCCAAGCTATTTCGG R: CGTCGGCAACAGTTTGAGTA	53	114–147
AIC5-25	(TG)_18_	F:CGATTCCCACCCTACCTAT R:AGACATGATTACGCCAGCTA	53	140–175
AIC5-39	(TG)_16_	F: TGGTGGTAAGAGGGTGAGTT R: TGCCAAGCTATTTAGGTGAC	51	150–181

Note: Tm, Annealing temperature; F, the forward primer; R, the reverse primer.

**Table 2:** The aquaculture harvest data of the nursery and adult stages of *A. i. concentricus*.

Stage	Site	Strain	Density	Shell Height (mm)	Shell Length (mm)	Shell Width (mm)	Body Weight (g)
Nursery	BH	Breeding	100	31.73 ± 3.40 ^Aa^	32.30 ± 3.68 ^Aa^	13.55 ± 1.59 ^Aa^	6.18 ± 1.96 ^Aa^
			200	29.10 ± 2.17 ^Ab^	29.61 ± 2.25 ^Ab^	12.35 ± 1.10 ^Ab^	4.64 ± 1.00 ^Ab^
			300	26.67 ± 2.95 ^Ac^	26.89 ± 3.10 ^Ac^	11.44 ± 1.38 ^Ac^	3.70 ± 1.09 ^Ac^
		Control	100	33.02 ± 2.43 ^Ba^	33.51 ± 3.71 ^Ba^	14.38 ± 1.69 ^Ba^	7.28 ± 2.18 ^Ba^
			200	28.93 ± 2.93 ^Ab^	29.16 ± 3.04 ^Ab^	12.58 ± 1.50 ^Ab^	4.88 ± 1.33 ^Ab^
			300	26.60 ± 2.60 ^Ac^	26.44 ± 2.76 ^Ac^	11.34 ± 1.23 ^Ac^	3.75 ± 1.23 ^Ac^
	QZ	Breeding	100	27.06 ± 2.46 ^Aa^	27.32 ± 2.73 ^Aa^	11.37 ± 1.29 ^Aa^	4.03 ± 1.10 ^Aa^
			200	24.73 ± 2.55 ^Ab^	24.55 ± 2.70 ^Ab^	11.30 ± 1.65 ^Aac^	2.99 ± 0.81 ^Ab^
			300	23.08 ± 2.58 ^Ac^	22.86 ± 2.72 ^Ac^	9.58 ± 1.22 ^Ac^	2.47 ± 0.82 ^Ac^
		Control	100	26.53 ± 3.32 ^Ba^	26.35 ± 3.58 ^Aa^	10.80 ± 1.81 ^Ba^	3.84 ± 1.84 ^Aa^
			200	24.36 ± 3.44 ^Ab^	24.15 ± 3.21 ^Ab^	9.92 ± 1.54 ^Ab^	3.34 ± 1.35 ^Bb^
			300	22.70 ± 2.74 ^Ac^	22.23 ± 2.86 ^Ac^	9.11 ± 1.27 ^Bc^	2.35 ± 0.84 ^Ac^
	FCG	Breeding	100	31.29 ± 3.30 ^Aa^	32.32 ± 3.78 ^Aa^	13.51 ± 1.73 ^Aa^	6.65 ± 2.06 ^Aa^
			200	29.19 ± 3.60 ^Ab^	29.78 ± 3.73 ^Ab^	12.62 ± 1.76 ^Ab^	5.32 ± 1.77 ^Ab^
			300	26.77 ± 2.90 ^Ac^	27.02 ± 3.21 ^Ac^	11.37 ± 1.47 ^Ac^	4.01 ± 1.09 ^Ac^
		Control	100	30.89 ± 3.06 ^Aa^	31.48 ± 3.33 ^Aa^	13.15 ± 1.59 ^Aa^	6.26 ± 1.80 ^Aa^
			200	27.96 ± 3.08 ^Bb^	28.07 ± 3.39 ^Ab^	11.52 ± 1.55 ^Bb^	4.48 ± 1.43 ^Bb^
			300	26.64 ± 3.29 ^Ac^	26.74 ± 3.51 ^Ac^	11.03 ± 1.72 ^Ac^	3.95 ± 1.47 ^Ac^
Adult	BH	Breeding	30	54.49 ± 2.38 ^Aa^	56.95 ± 2.62 ^Aa^	27.32 ± 1.02 ^Aa^	35.00 ± 2.78 ^Aa^
			45	51.99 ± 1.94 ^Ab^	54.10 ± 1.82 ^Ab^	26.71 ± 5.12 ^Ab^	29.61 ± 1.61 ^Ab^
			60	49.08 ± 1.83 ^Ac^	50.92 ± 2.07 ^Ac^	24.41 ± 1.23 ^Ac^	25.76 ± 2.03 ^Ac^
		Control	30	44.81 ± 1.71 ^Ba^	46.85 ± 2.22 ^Ba^	22.24 ± 1.21 ^Ba^	26.41 ± 5.09 ^Ba^
			45	42.30 ± 2.07 ^Bb^	43.47 ± 2.01 ^Bb^	20.72 ± 1.16 ^Bb^	22.49 ± 4.32 ^Bb^
			60	38.98 ± 2.02 ^Bc^	40.06 ± 2.36 ^Bc^	19.26 ± 1.05 ^Bc^	18.85 ± 4.09 ^Bc^
	QZ	Breeding	30	53.43 ± 2.91 ^Aa^	55.45 ± 2.96 ^Aa^	26.84 ± 1.56 ^Aa^	33.56 ± 3.59 ^Aa^
			45	51.40 ± 2.40 ^Ab^	53.13 ± 2.74 ^Ab^	26.07 ± 1.10 ^Ab^	30.02 ± 2.90 ^Ab^
			60	49.59 ± 2.33 ^Ac^	50.47 ± 2.48 ^Ac^	25.00 ± 1.11 ^Ac^	27.31 ± 3.98 ^Ac^
		Control	30	41.65 ± 2.44 ^Ba^	43.66 ± 2.85 ^Ba^	20.83 ± 1.68 ^Ba^	24.52 ± 6.12 ^Ba^
			45	40.06 ± 3.16 ^Bb^	41.67 ± 2.87 ^Bb^	20.11 ± 2.33 ^Bb^	21.20 ± 5.32 ^Bb^
			60	37.96 ± 3.66 ^Bc^	39.18 ± 2.89 ^Bc^	18.57 ± 1.51 ^Bc^	19.98 ± 5.34 ^Bc^
	FCG	Breeding	30	55.79 ± 2.13 ^Aa^	58.79 ± 2.18 ^Aa^	29.45 ± 1.74 ^Aa^	39.88 ± 3.59 ^Aa^
			45	54.39 ± 1.64 ^Ab^	56.62 ± 1.77 ^Ab^	27.86 ± 1.47 ^Ab^	35.48 ± 2.13 ^Ab^
			60	52.30 ± 1.68 ^Ac^	53.40 ± 5.49 ^Ac^	27.15 ± 1.57 ^Ac^	33.29 ± 3.53 ^Ac^
		Control	30	46.86 ± 1.92 ^Ba^	48.39 ± 1.85 ^Ba^	24.34 ± 1.82 ^Ba^	29.07 ± 5.22 ^Ba^
			45	44.54 ± 2.62 ^Bb^	46.37 ± 2.97 ^Bb^	23.15 ± 2.32 ^Bb^	27.28 ± 5.16 ^Bb^
			60	40.21 ± 2.31 ^Bc^	42.06 ± 2.32 ^Bc^	20.82 ± 1.36 ^Bc^	23.15 ± 5.91 ^Bc^

Note: Different superscript capital letters indicate significant differences (*p* < 0.05) among distinct strains at identical sites and stocking densities; different superscript lowercase letters denote significant differences (*p* < 0.05) among varying stocking densities for the same strain at identical sites.

**Table 3:** The mortality data of *A. i. concentricus* in different months during the adult culture stage.

Site	Strain	Density	Mortality (%)			
			2016-03	2016-04	2016-05	2016-06
BH	Breeding	30	0.56	1.11	2.78	10.00
		45	1.85	0.00	2.59	10.00
		60	1.11	1.94	1.11	10.56
	Control	30	1.11	0.56	1.67	5.56
		45	1.48	1.11	0.37	5.56
		60	1.39	0.28	1.11	8.06
QZ	Breeding	30	2.22	0.00	2.78	27.22
		45	0.74	1.11	1.85	22.22
		60	1.94	0.56	0.83	16.94
	Control	30	3.33	2.78	1.67	29.44
		45	1.85	1.85	1.85	17.78
		60	5.00	2.50	1.39	18.61
FCG	Breeding	30	3.89	0.56	1.67	9.44
		45	1.48	1.48	0.37	7.78
		60	1.39	1.11	1.94	6.11
	Control	30	2.78	0.00	0.56	6.67
		45	1.48	0.74	2.59	7.04
		60	2.78	0.00	1.39	6.94

**Table 4:** The phytoplankton genera and their dominance in the three culture sites.

Site	Genera	Average Dominance	Frequency of Occurrence (%)	Average Genera Abundance (cells∙L^−1^)
BH	*Coscinodiscus*	0.06	85.71	877
	*Thalassionema*	0.01	57.14	5607
	*Pleurosigma*	0.05	71.43	297
	*Skeletonema*	0.01	14.29	52,000
	*Melosira*	0.00	14.29	1
	*Ditylum*	0.00	14.29	144
	*Synedra*	0.00	71.43	262
	*Rhizosolenia*	0.01	71.43	1140
	*Pinnularia*	0.00	42.86	19
	*Odontella*	0.00	57.14	290
	*Cerataulina*	0.12	57.14	31,499
	*Chaetoceros*	0.28	100.00	60,613
	*Bacteriastraceae*	0.00	14.29	9
	*Hemiaulus*	0.00	14.29	29
	*Navicula*	0.03	71.43	124
	*Schroderella*	0.00	14.29	1
	*Nitzschia*	0.02	57.14	105
	*Corethron*	0.00	14.29	4
	*Licmophora*	0.01	71.43	61
	*Bacillaria*	0.01	28.57	267
	*Pseudo-nitzschia*	0.00	14.29	643
	*Leptocylindrus*	0.01	28.57	469
	*Planktoniella*	0.00	14.29	0
	*Triceratium*	0.00	14.29	7
	*Eucampia*	0.00	28.57	289
	*Asteroplanus*	0.00	14.29	436
	*Guinardia*	0.00	14.29	4
	*Ceratium furca*	0.08	85.71	173
QZ	*Coscinodiscus*	0.04	100.00	33
	*Fragilaria*	0.01	71.43	13
	*Thalassionema*	0.01	57.14	23
	*Pleurosigma*	0.25	100.00	210
	*Skeletonema*	0.03	71.43	46
	*Melosira*	0.04	71.43	57
	*Ditylum*	0.00	28.57	9
	*Synedra*	0.03	85.71	34
	*Rhizosolenia*	0.01	42.86	8
	*Pinnularia*	0.01	57.14	6
	*Cerataulina*	0.01	28.57	42
	*Chaetoceros*	0.01	28.57	38
	*Hemiaulus*	0.00	14.29	4
	*Navicula*	0.14	100.00	148
	*Schroderella*	0.00	14.29	2
	*Nitzschia*	0.05	100.00	43
	*Corethron*	0.00	14.29	1
	*Licmophora*	0.04	71.43	56
	*Gyrosigma*	0.01	71.43	9
	*Bacillaria*	0.06	85.71	60
	*Pseudo-nitzschia*	0.00	28.57	12
	*Leptocylindrus*	0.01	28.57	22
	*Fragilariopsis*	0.00	14.29	0
	*Gossleriella*	0.00	14.29	2
	*Thalassiosira*	0.00	14.29	2
	*Stephanopyxis*	0.00	14.29	45
	*Guinardia*	0.00	14.29	4
	*Ceratium furca*	0.00	57.14	2
	*Phormidiaceae*	0.00	14.29	0
FCG	*Coscinodiscus*	0.04	100.00	163
	*Fragilaria*	0.01	57.14	49
	*Thalassionema*	0.15	100.00	3784
	*Pleurosigma*	0.03	100.00	248
	*Skeletonema*	0.04	57.14	480
	*Melosira*	0.00	14.29	17
	*Ditylum*	0.00	57.14	80
	*Synedra*	0.03	85.71	888
	*Rhizosolenia*	0.05	57.14	413
	*Pinnularia*	0.01	100.00	81
	*Odontella*	0.00	71.43	19
	*Cerataulina*	0.06	57.14	1008
	*Chaetoceros*	0.29	85.71	38,666
	*Bacteriastraceae*	0.00	14.29	0
	*Hemiaulus*	0.00	42.86	79
	*Navicula*	0.01	85.71	115
	*Nitzschia*	0.00	71.43	176
	*Gyrosigma*	0.00	14.29	6
	*Bacillaria*	0.00	14.29	157
	*Pseudo-nitzschia*	0.00	28.57	67
	*Leptocylindrus*	0.00	28.57	414
	*Fragilariopsis*	0.00	42.86	153
	*Detonula*	0.00	28.57	183
	*Thalassiosira*	0.00	14.29	23
	*Eucampia*	0.00	14.29	204
	*Guinardia*	0.01	28.57	416
	*Ceratium furca*	0.01	100.00	68

Note: Frequency of occurrence refers to the percentage of observed occurrences relative to the total number of observations.

**Table 5:** Microsatellite-based genetic variability parameters of six scallop populations.

Populations	Average *N_A_*	Aveage *N_E_*	Average *H_O_*	Average *H_E_*	Average *PIC*
BH-B	2.80	2.13	0.37	0.49	0.41
QZ-B	2.80	1.82	0.42	0.44	0.37
FCG-B	3.70	2.07	0.42	0.53	0.46
BH-C	4.40	3.04	0.38	0.62	0.55
QZ-C	3.70	1.98	0.46	0.48	0.42
FCG-C	4.20	2.61	0.40	0.62	0.55

Note: *N_A_*, the allele number; *N_E_*, effective allele number; *H_O_*, observed heterozygosity; *H_E_*, expected heterozygosity; *PIC*, Polymorphism information content; B, Breeding population of *A. i. concentricus*; C, Control population of *A. i. concentricus*.

## Data Availability

Data will be made available on request.
